# Successful Fertilization in a Case of Globozoospermia Through Assisted Oocyte Activation With Calcium Ionophores: A Case Report

**DOI:** 10.7759/cureus.61065

**Published:** 2024-05-25

**Authors:** Priyanka Thapa, Pranita A Bawaskar, Ankit Badge, Charu Pareek, Avanti Kalbande

**Affiliations:** 1 Clinical Embryology, Datta Meghe Medical College, Datta Meghe Institute of Higher Education and Research (DU), Nagpur, IND; 2 Microbiology, Datta Meghe Medical College, Datta Meghe Institute of Higher Education and Research (DU), Nagpur, IND; 3 Obstetrics and Gynaecology, School of Allied Health Sciences, Datta Meghe Institute of Higher Education and Research (DU), Wardha, IND

**Keywords:** in vitro fertilization, assisted oocyte activation, calcium ionophore, intracytoplasmic sperm injection, infertility

## Abstract

Globozoospermia is a rare sperm morphological abnormality characterized by a lack of acrosomes and post-acrosomal sheaths, defects in the cytoskeleton around the nucleus, and separated nuclear membranes. In this case, the study outlines the treatment of a 32-year-old male patient diagnosed with globozoospermia. The couple, facing primary infertility for seven years, had already undergone unsuccessful assisted reproductive technology treatments, such as two intrauterine inseminations and one in vitro fertilization. They opted for intracytoplasmic sperm injection (ICSI) with assisted oocyte activation (AOA) using a calcium (Ca) ionophore. The semen analysis showed globozoospermia, which indicated that ICSI was required for fertilization. Post-fertilization, embryo quality was assessed; three were in cleavage-stage embryos, and two grade 4AA and 3AA blastocysts and the rest were arrested at 2 pronuclear (2PN) stages, revealing successful embryo development. This case report implies that using AOA with Ca ionophores enhanced the fertilization outcomes and could be a helpful intervention strategy for patients with globozoospermia.

## Introduction

Infertility is the "inability of couples to have a baby after one year of regular unprotected intercourse” [1]. Infertility is a significant global health concern that impacts around 8% to 10% of couples worldwide [2,3]. Male infertility can be caused by various factors, including testicular dysfunction, endocrine disorders, lifestyle choices such as alcohol consumption, smoking, tobacco use, and drug use, congenital anatomical factors, and exposure to genotoxic substances, which can lead to reduced fertility or infertility. To diagnose male infertility, a complete clinical history, physical checkup, and analysis of semen are required [4]. A female who has never conceived before is primary infertility, while secondary infertility is the inability of a couple to conceive after at least one prior successful conception [5].

Globozoospermia is a rare disorder of sperm morphology characterized by round-headed spermatozoa without acrosome, cytoskeleton defects around the nucleus, absence of a post-acrosomal sheath, and the separation of nuclear membranes [6]. The pathogenesis of globozoospermia most probably originates during spermiogenesis, particularly during acrosome formation and sperm head elongation [7]. A partial globozoospermia is defined by a variable percentage between 20% and 90% of spermatozoa with abnormal morphology. In contrast, total globozoospermia is characterized by a completely round-headed sperm that is unable to penetrate the oocyte, causing primary infertility. The incidence of globozoospermia is less than 0.1% [8,9]. Due to the absence of the acrosome, the spermatozoa cannot penetrate the zona pellucida [10]. The main proteinase found in the mature sperm's acrosome is known as acrosin. When stimulated, the acrosome discharges its contents into the zona pellucida. After the reaction, the zymogen form of the protease enzyme transforms into its active form, which is called β-acrosin. The active enzyme promotes the penetration of the sperm through the inner layers of glycoproteins of the ovaries by lysing the zona pellucida [11].

Oocyte activation (OA) is a controlled process that begins when sperm and oocytes fuse, causes intracellular calcium (Ca) oscillations, and activates oocytes [12]. Assisted oocyte activation (AOA) methods include electrical, mechanical, and chemical activation. Among these methods, the chemical AOA method using Ca ionophores is the most widely used and effective technique. This method increases the intracellular Ca level and recruits the same from the culture medium outside the oocyte, activating oocytes more efficiently [13]. AOA is a method that imitates the natural signaling mechanisms in cells to activate oocytes. In humans, Ca ionophore A23187 is the most commonly used AOA method [14]. Due to its successful results, Ca ionophore has become a choice in many medical facilities. However, it is essential to note that Ca oscillation is linked to the paternal genome's active and rapid demethylation of the maternal genome and the passive deoxyribonucleic acid (DNA) demethylation. Hence, it is acceptable to suggest that changes in genomic imprinting may be related to altered Ca signals, which have been reported following Ca ionophore treatment [15]. In this case study, we aim to increase the fertilization rate by using Ca ionophores in patients with globozoospermia.

## Case presentation

Patient information

In 2023, the infertile couple with primary infertility who had been trying to conceive for the past seven years started their assisted reproductive technology (ART) treatment at Wardha Test Tube Baby Centre (WTTBC) in Maharashtra, India. The female was 29 years old, and the male was 32 years old. During a routine semen examination, the diagnosis of globozoospermia was made. Both provided clear and detailed information on all procedures, advantages, and disadvantages and informed consent was obtained through consultation.

Clinical history

The couple had a history of two failed intrauterine insemination (IUI) cycles and one failed in vitro fertilization (IVF) cycle, where a total of 12 oocytes were retrieved. Unfortunately, there was a fertilization failure since none of the retrieved oocytes fertilized. The male patient had a history of smoking and alcohol consumption for eight years. The female patient did not have any addictions; neither male nor female had any past surgery or medical history in their family. The couple had no medical history of cardiac issues, tuberculosis, or hypertension and no prior history of psychological distress.

Clinical findings

The male and female patients' overall physical health and vitals were within the normal range. The male patient exhibited typical secondary sexual characteristics, suggesting adequate testosterone production. The palpation of the scrotum revealed the presence, size, position, and shape of the testicles, which appeared normal. During physical examination, the patient's testicular volume was within the normal range (15 mL-35 mL), with the right testicle measuring 25 mL and the left measuring 24 mL. The female patient's menstrual cycle was regular ±28 days, and blood tests were performed to assess hormonal levels. These tests included anti-Müllerian hormone (AMH), follicle-stimulating hormone (FSH), and luteinizing hormone (LH), which were found to be within the normal range. The male patient was advised to undergo semen analysis and blood tests to assess the semen parameters and hormonal profile, and the total sperm concentration was evaluated by Makler counting chamber. The patient's sperm count was 33 million/ml, and the total motility was 60%, with progressive motility was 32%, pH was 7.5, and volume was 2 ml, which was standard in range, but 97% of sperm morphology was found to be globozoospermia. Table 1 shows the semen analysis report.

**Table 1:** Semen analysis. ml: Millimeters; pH: potential of hydrogen; PR: progressive motility; NP: non-progressive motility

Semen parameters	Results	Reference value
Ejaculatory abstinence	2 days	2-7 days
Volume	2 ml	1.4ml
Appearance	Opalescent grey	Opalescent grey
pH	7.5	7.2-8.0
Total sperm count	33 million/ml	16 million/ml
Total sperm motility (PR+NP)	60 %	42%
Progressive motility	32%	30%
Morphology	97%	>4%

The hormonal profile of the male patient results revealed that all hormonal levels were normal, including testosterone levels, which was 1.7 mIU/mL within the normal range, and no sign of hypogonadism was observed. The hormonal profile results are shown in Table 2.

**Table 2:** Results of the male hormonal profile. AMH: Anti-Müllerian hormone; FSH: follicle-stimulating hormone; LH: luteinizing hormone; ng/mL: nanograms per milliliter; mIU/mL: milli-international units per milliliter

^Hormone^	^Findings^	^Reference range^
^Serum AHM^	^4.1 ng/mL^	^1.0-40 ng/mL^
^FSH^	^12.1 mIU/mL^	^3.5-12.5 mIU/mL^
^LH^	^11 mIU/mL^	^2-15 mIU/mL^
^Testosterone^	^1.7 mIU/mL^	^0.8-7.6 mIU/mL^

**Figure 1:**
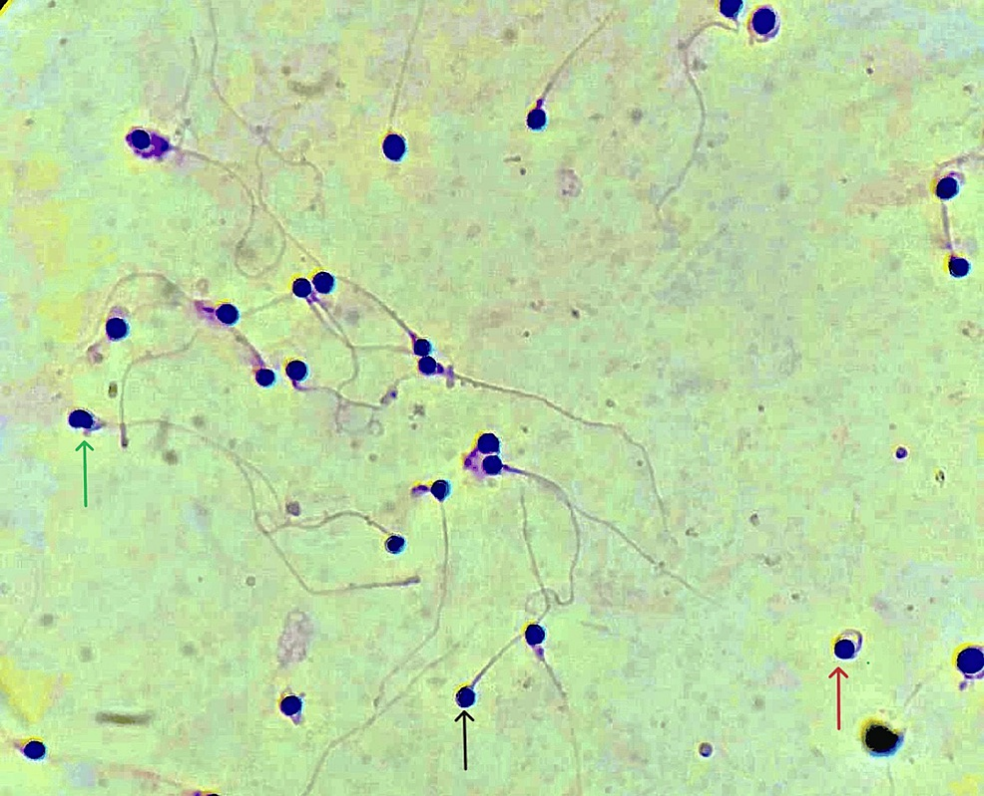
A microscopic view of PAP stained spermatozoa. All blue round structures depict globozoospermia; the black arrow shows a straight flagellum with round-headed spermatozoa with no perinuclear cytoplasmic content; the red arrow shows coiled flagellum; the green arrow shows nuclear separation PAP: Papanicolaou

Papanicolaou (PAP) staining was done to evaluate morphological abnormalities, and at least 200 spermatozoa were examined per slide. A sperm with a round head, absence of an acrosome, a post-acrosomal sheath, defects around the nucleus, and separation of nuclear membranes are shown in Figure 1.

Diagnosis

The diagnosis was established based on the semen analysis. The semen analysis confirmed a globozoospermia and had 97% round-head sperm indicating that the male factor is the leading cause of infertility.

Therapeutic intervention

The preparation for oocyte retrieval for the female patient was initiated using an antagonist protocol to stimulate ovaries. The gonadotropin hormone-releasing (GnRH) hormone antagonist was used on the third day of the menstrual cycle by combining 150 international units (IU) of FSH with 2.5 mg/d of the GnRH antagonist until the follicles reached 14-16 mm in diameter. After the follicle reached an average diameter of 16mm, 10,000 IU of human chorionic gonadotropin (hCG) was administered on day 10 of the menstrual cycle. Ovum pick-up (OPU) was performed 36 hours after hCG administration. A total of 14 oocytes were retrieved, including one germinal vesicle stage oocyte, two metaphase I (MI) oocytes, and 11 metaphase II (MII) oocytes. For the male patient, fresh ejaculate was collected by masturbation following 2-7 days of abstinence. The semen sample was allowed to liquefy at 37°C for at least 15 minutes. After liquefaction, the swim-up technique involved placing the semen in a sterile 15-ml conical centrifuge and overlaying it with a supplemented medium in a 1:2 ratio. The sample was centrifuged at 1000-1200 revolutions per minute (rpm) for 10-12 minutes, and the supernatant was discarded. The pellet was gently overlayered with the supplemented medium again, and the tube was inclined at 45° and kept at 37°C for 30-40 minutes. Then, 0.5 ml supernatant was used to perform further procedures. Following the preparation, the semen sample showed 29 million/ml sperm concentration in 1.8 ml volume with 50% total motility with 30% progressive motility and pH of 7.4, morphology suggesting globozoospermia (95%).

Soon after the OPU, the oocytes were denuded, followed by intracytoplasmic sperm injection (ICSI). Subsequently, the resulting embryos were transferred to a medium containing Ca ionophore for further development. Embryos were incubated for one hour in the same medium and then transferred to a culture medium, explicitly utilizing a one-step culture medium approach. After this incubation period, fertilization was assessed approximately 16-18 hours post-ICSI. Subsequently, embryonic development assessments were conducted post-fertilization on days three and five. All 11 MII oocytes were fertilized, resulting in three cleavage-stage embryos and two blastocysts of grade 4AA and 3AA, rest got arrested into 2 pronuclear (2PN) stage, as per the Gardner grading system [16]. After the fresh embryo transfer (ET), the outcome was monitored by assessing β-human chorionic gonadotropin (β-hCG) levels. 

Follow-up

The fresh ET process was done to implant two blastocysts with grades 4AA and 3AA, respectively. After 16 days post-ET, a urine pregnancy test revealed a positive result. Further serum β-hCG levels were assessed with 1212 mIU/mL levels, confirming the pregnancy. Additionally, ultrasound sonography was done during the third week of gestation, revealing the presence of a single sac formation.

## Discussion

This was a case of globozoospermia in which the couple had faced two unsuccessful IUIs and one IVF failure in the past. We assessed the impact of Ca ionophore-aided OA post-ICSI on fertilization rates and embryo quality in patients. Individuals with globozoospermia face infertility due to their sperm's incapability to attach to the zona-pellucida and enter the oocyte. Consequently, resorting to ICSI becomes the sole viable option for achieving pregnancy. Nevertheless, the fertilization rate post-conventional ICSI is suboptimal, indicating that, beyond the incapability to penetrate the zona pellucida, other essential procedures are deficient in these patients. Notably, a pivotal role appears to be played by the absence of factors linked to OA found in the acrosome [17]. In agreement with the author, our patient faced failed IUIs and IVF cycles without any interventional treatment. Globozoospermia affected the fertilization rates in the previous IVF cycle even after performing ICSI. Therefore, Ca ionophore treatment was given to facilitate fertilization in the embryos.

The absence of OA is the primary reason for the lack of successful fertilization following traditional ICSI. To transform an MII-arrested oocyte into a fertilized ovum, a sequence of processes is involved by OA. In mammalian oocytes, a surge in intracellular Ca levels, initiated shortly after the fusion of spermatozoon and oocyte, is responsible for restarting meiosis and initiating embryo development. In some men experiencing fertilization challenges, such as those with globozoospermia, their spermatozoa either completely fail to induce sufficient Ca oscillations upon injection into oocytes or elicit Ca oscillations with reduced frequency and amplitude compared to sperm from fertile men [6]. Given a history of infertility spanning seven years and previous unsuccessful attempts with ICSI, the introduction of post-ICSI treatment with Ca ionophores proved to be a beneficial intervention in our case. The fertilization rate significantly improved compared to the outcome of the prior ICSI procedure conducted without Ca treatment.

The amount of intracellular Ca is significantly increased by adding Ca ionophores, which promotes the transfer of Ca from extracellular membranes to cell membranes. Thus, inadequate OA is the main reason for unsuccessful ICSI fertilization [18]. In our study, it has been found that using Ca ionophores can increase the rate of fertilization and improve embryo development. However, it is necessary to realize that early environmental factors might impact embryo development and cause chromosomal abnormalities or growth inhibition.

## Conclusions

AOA, by utilizing Ca ionophores, can enhance fertilization rates and embryo quality, in individuals diagnosed with globozoospermia. This case report suggests that ICSI with AOA resulted in successful embryo development and positive pregnancy. Thus, using Ca ionophores post-ICSI in the case of globozoospermic patients can be one of the methods for treating male factor infertility. This method has improved fertilization rates and embryo quality in patients with globozoospermia, leading to pregnancy. To determine its effectiveness and potential advantages, further research should be conducted using ionophores post-ICSI in these patients.
